# An Observing System Simulation Experiment for the Western North Pacific Region

**DOI:** 10.1155/2014/314134

**Published:** 2014-11-09

**Authors:** Shuhei Masuda

**Affiliations:** Research and Development Center for Global Change, Japan Agency for Marine-Earth Science and Technology (JAMSTEC), Natsushima 2-15, Yokosuka, Kanagawa 237-0061, Japan

## Abstract

This study investigated the effectiveness of concentrated observations for ocean state estimation in a region remote from the observation site. I executed a twin observing system simulation experiment (OSSE) for the North Pacific region, using an ocean data synthesis system, to examine how the potential effectiveness is for a well-defined criterion, the representativeness of the subsurface salinity minimum corresponding to North Pacific Intermediate Water (NPIW). The results of the OSSE show that data synthesis confined to the region corresponding to the recent origin of the NPIW (35°N–53°N, 130°E–170°E) can affect the modeled extent of the NPIW in the central Pacific at 35°N, 180°. The interannual variability of the NPIW is not well reproduced in terms of the standard deviation value (std), only by the data input in the origin region. The root mean square difference between the “true” and the synthesized field is twice larger than the std although there the representativeness of the scale of salinity minimum is improved by about one-third of the difference between the “true” and “first-guess” fields in a snapshot. These results imply that combinations of concentrated and other in situ observations should be required for the dynamic state estimation of the NPIW.

## 1. Introduction

Ocean state estimation based on a synthesis of observational data and model results is a powerful approach to better understand climate change, particularly with the use of a smoothing method [1]. Developments in computer science have made long-term state estimates of the global ocean a tractable problem [2]. Observations are among the most important factors determining the quality of an ocean state estimate, but global ocean observations are limited by practical considerations such as the cost of deploying observation instruments. Subject to such constraints, an effective ocean observing system is required to support climate change research.

Köhl and Stammer [3] have shown that an adjoint sensitivity analysis is useful for determining the optimal observing system for a regional sea. Their scheme is promising for application to the global ocean. Heimbach et al. [4] have clearly demonstrated that adjoint sensitivity analysis is useful for observing system design for a large scale ocean circulation research. A twin oceanic observing system simulation experiment (OSSE) also is a useful approach for evaluating new observing systems or alternative deployment strategies for existing systems [5]. Recently, Halliwell et al. [5] carefully constructed one such OSSE system in a domain of the Gulf of Mexico and obtained credible observing system impact assessment.

In this paper, I report results of a twin OSSE to examine the effect of concentrated observations within a limited region, specified on the basis of an adjoint sensitivity analysis, on the estimated basin-scale ocean state. This study focused on estimating salinity in North Pacific waters in conjunction with the North Pacific Intermediate Water (NPIW) [6]. The properties of the NPIW are closely related to cross gyre processes [7–9], and they also influence the formation of vertical stratification in conjunction with ocean circulation and play an important role in determining the meridional mass transports in the middle layer. Long-term changes in this water have been focused on and examined by Nakano et al. [10]. My focus is, in particular, interannual variability of the NPIW. The goal of this report is to demonstrate how the observations in a “key” region defined by an adjoint sensitivity analysis applied to the NPIW can potentially affect the representation of that water in a decadal state estimation.

## 2. Methods

### 2.1. Ocean Data Synthesis System and Adjoint Sensitivity Analysis

The ocean data synthesis system was developed as part of the Japan Agency for Marine-Earth Science and Technology- (JAMSTEC-) Kyoto University collaborative program (the K7 consortium). The ocean general circulation model (OGCM) applied to this system is based on version 3 of the Geophysical Fluid Dynamics Laboratory (GFDL) Modular Ocean Model (MOM) [12]. The horizontal resolution is 1° in both latitude and longitude, and there are 46 vertical levels for the global ocean basin. The data synthesis method is based on a four-dimensional variational (4D-VAR) approach, with adjoint code from the global OGCM. This system, described in detail elsewhere [11, 13, 14], seeks the optimal solution (the best temporal trajectory of the model results) that synthesizes the OGCM results and observational data within the framework of the model formalism and thus provides a dynamically self-consistent dataset. I used this system to carry out a twin OSSE by changing the observational elements.

Adjoint sensitivity analysis yields the temporal rate of change of a physical variable at a fixed point in time and space when model variables are arbitrarily changed in the four-dimensional continuum of one temporal and three spatial coordinates. This is equivalent to specifying the sensitivity of a variable to small perturbations of the parameters governing the oceanic state [15–17]. The estimate of the adjoint solution expresses the system's sensitivity to fluctuations of model variables, which can aid in identifying and characterizing the origins and pathways of specific water masses. I used the results of a previous adjoint sensitivity analysis for the NPIW [11] to define the region for testing a more closely focused “virtual” set of observations.

### 2.2. Observing System Simulation Experiment

Masuda et al. [11] performed a sensitivity calculation in which they investigated the sensitivity of salinity to a delta-function perturbation of salinity in the vicinity of the NPIW at 43°N, 180° at a depth of 400 m (approximately 26.8σ*θ*; their Figure 4). The recent sources of this water can be traced as far as 3 years back in time, mainly to the east of Japan along the Kuroshio Extension and the North Pacific Current (their Figure 4(b)), with a smaller contribution from the western subarctic gyre region.

The results of this sensitivity analysis motivated the twin OSSE described in this paper. To reveal the influence of data input in a limited region on the representativeness of the NPIW in a state estimate, I conducted an identical twin experiment in which an optimized ocean state, obtained by the synthesis of data from all available observations, was used as the “true” state.

**Figure 1:**
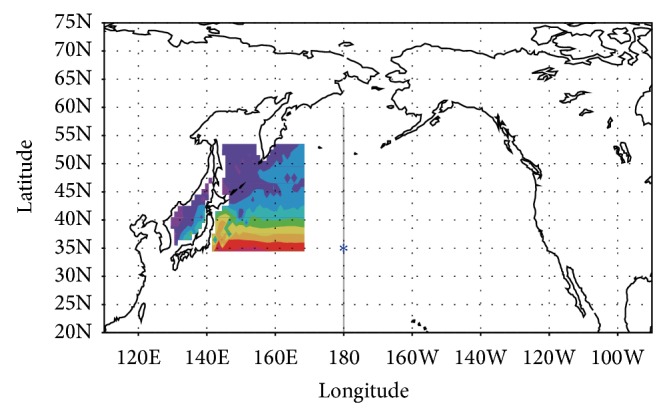
Subsampling region (color) adopted as the result of an adjoint sensitivity analysis [11]. Color shading is the typical temperature pattern at a depth of 300 m in this region. The blue asterisk denotes the point where the remote effect of changes in the subsampled data was evaluated (see Section 3 for details). The representativeness of the vertical structure of the salinity field was assessed along the gray line at 180° (see Figure 4).

Two comparative ocean state estimates were carried out by subsampling input data from the true state. The first, “analysis” case, was an ocean state estimate that used water temperature and salinity data from the true state only in the “recent origin” area from surface to bottom ((35°N–53°N, 130°E–170°E; color shading in Figure 1) as virtual concentrated observations. The second, first-guess case, was a simulation result that did not incorporate observations. The assimilation window was 10.5 years from January 1990 to June 2000. The period is arbitrarily selected. The length of window depends on available computational resources. The observational errors and representativeness errors were assumed to be the same as those applied in the long-term state estimation experiment [13]. This corresponds to an assumption that monthly mean values for temperature and salinity are obtained within the area every 1 degree in both latitude and longitude as so-called super-observation with the same quality of recent ocean observations. Other aspects of the ocean state estimate were also the same as those used by Masuda et al. [13].

## 3. Results

**Figure 2:**
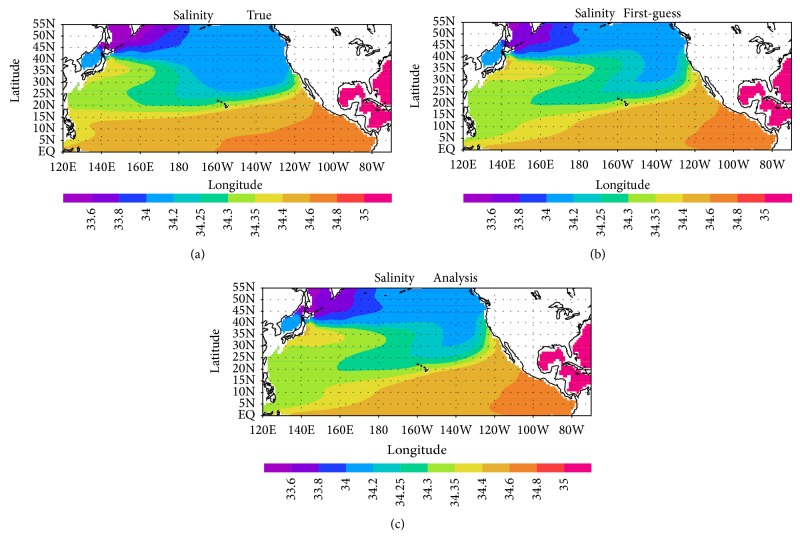
Distribution of salinity on the 26.8σ*θ* isopycnal surface averaged over 1995–2000: (a) true simulation for the twin experiment, (b) first-guess simulation with no observational input, and (c) analysis simulation in which the virtual concentrated observations were synthesized.

Figure 2 shows the mean spatial distribution of salinity on the 26.8σ*θ* isopycnal surface during 1995–2000. The data synthesis with the virtual concentrated observations enables the system to better represent the NPIW, which is characterized by a salinity minimum (34.2), in particular, along the 35°N–40°N latitude band (Figures 2(b) and 2(c)). However, it does not perfectly reproduce the true state (Figure 2(a)) since there is lack of information from observations in other regions except for the recent source region (Figure 1), inclusive of in situ observations. Hereafter, I investigate the influence of the virtual observations on the representativeness of the subsurface water properties.

**Figure 3:**
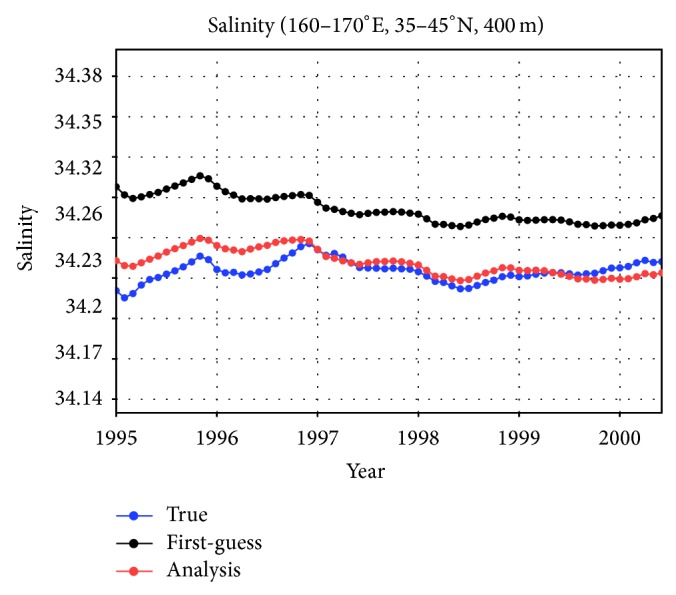
Time series of salinity values at 400 m depth (closely corresponding to the 26.6σ*θ* isopycnal surface) averaged over the region bounded by 35°N–45°N and 160°E–170°E, within the region where virtual concentrated observational data were synthesized (Figure 1).

The data synthesis reproduced the time series of salinity values at 400 m depth averaged over the region bounded by 35°N–45°N and 160°E–170°E, part of the area where virtual observations were subsampled (Figure 3). The system corrected the subsurface salinity values by about 0.04 from the first-guess results (without data input) during the simulation period. The standard deviation value (std) for the monthly mean time series is 9.7 × 10^−3^ for the analysis case. The root mean square difference between the true and analysis cases has a smaller value of 8.8 × 10^−3^ than the std, which shows that the analysis field is broadly acceptable in this region. In addition, the correlation coefficient value between the true and analysis cases is 0.50 within a 99.5% level of confidence. Note that this correction is not necessarily obvious when a 4D-VAR data assimilation approach is being used, but it is desirable.

**Figure 4:**
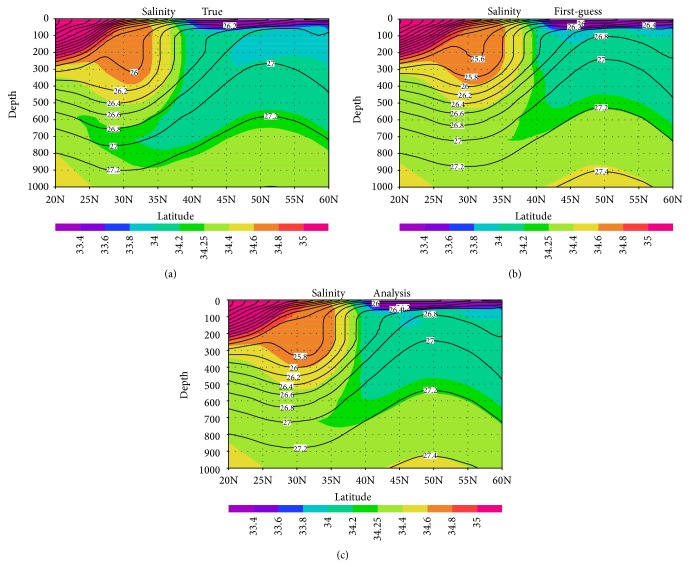
Mean salinity distribution (colors) and contours of potential density (σ*θ*) in a vertical cross section along 180° (gray line in Figure 1) averaged over 1995–2000: (a) true case, (b) first-guess case, and (c) analysis case.

Here we assess the properties of the simulated NPIW in a region remote from the virtual observations. Figure 4 shows the mean salinity distribution in a vertical cross section along 180°. The NPIW, characterized by the salinity minimum around 34.2, extends south of 35°N in the true state and is well defined (Figure 4(a)). In the analysis state, a salinity minimum of 34.2 (34.25) extends south of 40°N (35°N) (Figure 4(c)). Although the NPIW is by and large indistinct compared with its true state, the feature is confirmed to be certainly improved by the input of virtual observations that are distant from 180° (Figures 4(b) and 4(c)).

**Figure 5:**
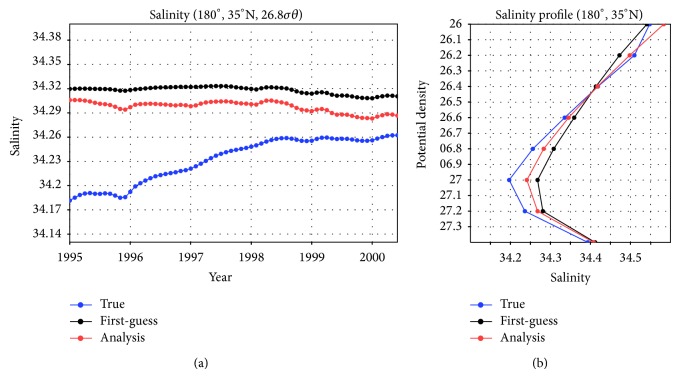
(a) Time series of salinity values on the 26.8σ*θ* isopycnal surface at 35°N, 180° (asterisk in Figure 1), a point remote from the region of synthetic concentrated observations. (b) Vertical salinity profiles at 35°N, 180° from 26.0 to 27.4σ*θ* in January 2000.

In the time series of salinity values on the 26.8σ*θ* isopycnal surface at a remote location (35°N, 180°) where no observations were subsampled, the values in the analysis case are proven to approach the true values (blue and red curves in Figure 5(a)). Quantitatively, in comparison to the std of 2.8 × 10^−2^ for the true case, that for the analysis case is 6.6 × 10^−3^. There seems to be significant difference since the root mean square difference between the two time series has a value of 6.5 × 10^−2^, twice the std for the true case. Only the data input in the recent origin region is thus not enough to reproduce the temporal changes in the NPIW in a state estimation with a decadal assimilation window.

Vertical salinity profiles at this position (Figure 5(b)) show that the value of the salinity minimum is improved by about one-third of the difference between the true and first-guess fields in this well-defined case.

The concentrated observations in the recent origin, detected by an adjoint sensitivity, have thus limited influence on the representation of the water in the synthesis system. This result implies that some combination of concentrated observations and other in situ observations is required in the case of NPIW monitoring.

## 4. Conclusion and Discussion

In this report, I clarify how the potential influence of a proposed radical observing system for the NPIW is by OSSE. Although the specific values obtained from this demonstrative study may depend on the details of the model platform, the data synthesis approach taken, and the target period chosen, this study demonstrates the possible effectiveness/limit of concentrated observations in the western North Pacific on the ocean state estimate for remote regions, in conjunction with the NPIW. The results imply that observations from a limited region of the NPIW origin require some additional in situ information for better representation of the NPIW in state estimates.

In this study, a set of observations with a uniform quality (as super-observation) is only considered in the OSSE. In contrast, there are many varieties of measurements inclusive of CTDs, XBTs, Argo profiling floats, remote sensing satellite, and so on. They provide exhaustive information from the very surface to the deepest layers of the global ocean. Further work considering the synergetic effects of such information from various instruments will enable us to identify the more effective and more practical requirements for the future observing systems.
